# Supplementary material for: Nitrogen-Doped Carbons Derived from Imidazole-Based Cross-Linked Porous Organic Polymers
Source: Molecules. 2021 Jan 27;26(3):668. doi: 10.3390/molecules26030668 (PMC7865342; doi:10.3390/molecules26030668)
Supplement: Supplementary file 1 [file molecules-26-00668-s001.pdf]

Electronic Supplementary Materials for:

Nitrogen-Doped Carbons Derived from Imidazole-Based Cross-Linked Porous Organic Polymers

Wojciech Kiciński*, Sławomir Dyjak

Institute of Chemistry, Military University of Technology, 2 Kaliskiego Str., PL 00-908 Warsaw, Poland

***Corresponding author. E-mail address:** wojciech.kicinski@wat.edu.pl (W. Kiciński)

Syntheses of organic and carbon gels

1. **CN: Imidazole-2-carboxaldehyde-based gels**

1.83 g of imidazole-2-carboxaldehyde and 0.80 g of phloroglucinol (molar ratio of 3:1), were dissolved in a mixture of 15 ml DMF and 6 ml isopropanol. A black solution was obtained upon heating to ca. 50-60 °C. The solution was acidified with 1.0 ml of concentrated hydrochloric acid. Upon acidification a wet solid gel was obtained instantaneously. Upon aging at 55 °C for 24 h (in a tight container) the gel was immersed in acetone to replace DMF and then dried at 70 °C for 24 h. Eventually 2.84 g of organic gel was obtained. The dried gel was subjected to pyrolysis under N_2.

Pyrolysis at 750 °C – yield = 40%

Pyrolysis at 850 °C – yield = 36%

Pyrolysis at 950 °C – yield = 34%

2. **<u>CN(M): 1-Methylimidazole-2-carboxaldehyde-based gels</u>**

2.10 g of 1-methylimidazole-2-carboxaldehyde and 0.80 g of phloroglucinol (molar ratio of 3:1) were dissolved in a mixture of 15 ml methanol and 6 ml isopropanol. Then the solution was acidified with 1.0 ml of concentrated hydrochloric acid. Upon acidification a wet solid gel was obtained after ca. 15 min. Upon aging at 55 ^{o}C for 24 h (in a tight container) the gel was dried at 70 ^{o}C for 24 h. Eventually 2.92 g of organic gel was obtained. The dried gel was subjected to pyrolysis under N_2.

Pyrolysis at 750 °C – yield = 33%

Pyrolysis at 850 °C – yield = 29%

Pyrolysis at 950 °C – yield = 27%

3. **<u>CNS: 2-Thiazolecarboxaldehyde-based gels</u>**

2.15 g (1.70 ml) of 2-thiazolecarboxaldehyde and 0.80 g of phloroglucinol (molar ratio of 3:1) were dissolved in a mixture of 15 ml methanol and 6 ml isopropanol. Then the solution was acidified with 1.0 ml of concentrated hydrochloric acid. Upon acidification a wet solid gel was obtained after ca. 15 min. Upon aging at 55 °C for 24 h (in a tight container) the gel was dried at 70 °C for 24 h. Eventually 2.64 g of organic gel was obtained. The dried gel was subjected to pyrolysis under N_2.

Pyrolysis at 750 °C – yield = 35%

Pyrolysis at 850 °C – yield = 33%

Pyrolysis at 950 °C – yield = 30%

Table S1. Elemental analysis (wt.%) and textural characteristics of the organic and carbon gels obtained from a phloroglucinol/aldehyde mixture of 1:2 molar ratio.

Sample	N (%)	C (%)	H (%)	S (%)	S_{BET} (m^2g^{-1})	V_t (cm^3g^{-1})
**CN**	15.55	44.46	4.94	-	-	-
**CN–750**	10.61	77.72	1.62	-	490	0.20
**CN–850**	8.09	75.71	1.81	-	500	0.22
**CN–950**	6.64	88.52	0.57	-	510	0.23
**CN(M)**	14.69	44.82	5.24	-	-	-
**CN(M)–750**	9.01	77.94	1.72	-	517	0.23
**CN(M)–850**	7.10	78.33	1.70	-	510	0.22
**CN(M)–950**	5.39	89.87	0.53	-	497	0.21
**CNS**	7.14	43.25	3.43	15.77	-	-
**CNS–750**	5.27	77.74	1.31	7.16	680	0.28
**CNS–850**	4.30	79.36	1.18	5.71	705	0.29
**CNS–950**	3.56	85.81	0.72	4.49	765	0.33

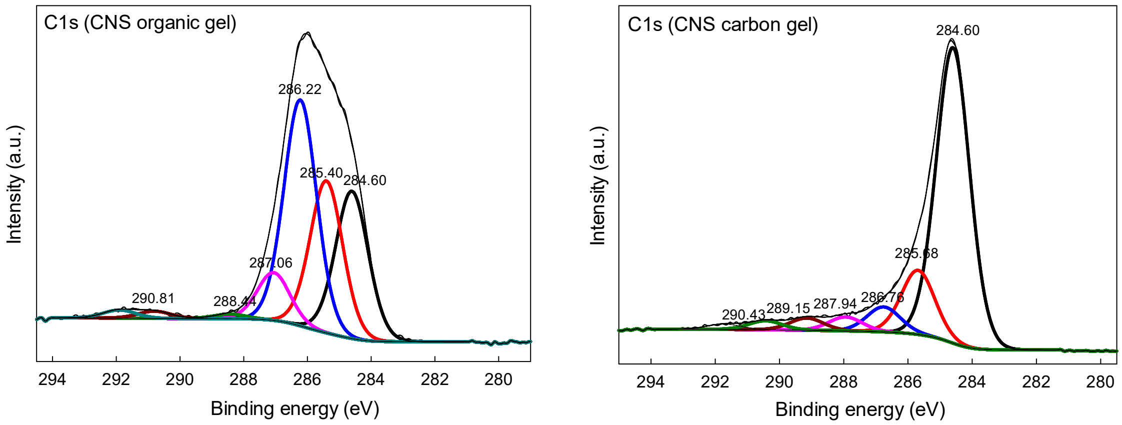

Figure S1. High resolution XPS spectra of C 1s of organic and corresponding carbon gels obtained from 2-thiazolecarboxaldehyde via pyrolysis at 750 °C.

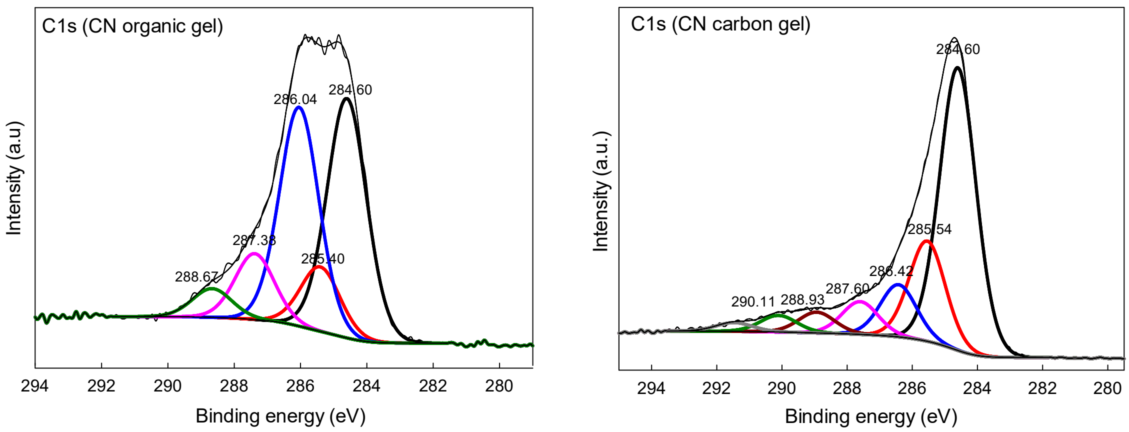

Figure S2. Comparison of high resolution XPS spectra of C 1s of organic and corresponding carbon gels obtained from imidazole-2-carboxaldehyde via pyrolysis at 750 °C.

Table S2. Elemental surface composition of the N-doped and N,S co-doped samples (the organic and carbon gels obtained from a phloroglucinol/aldehyde mixture of 1:3 molar ratio) based on the survey XPS spectra.

**Sample**	**Elemental surface composition (at.%)**				
	**C**	**N**	**O**	**S**	**Cl (from HCl)**
**CNS (organic polymer)**	67.4	7.9	16.0	8.0	0.7
**CN (organic polymer)**	64.4	10.5	20.7	0	2.6
**CNS–750**	90.7	3.3	2.0	3.6	0
**CN–750**	77.7	7.8	9.6	0.4	0

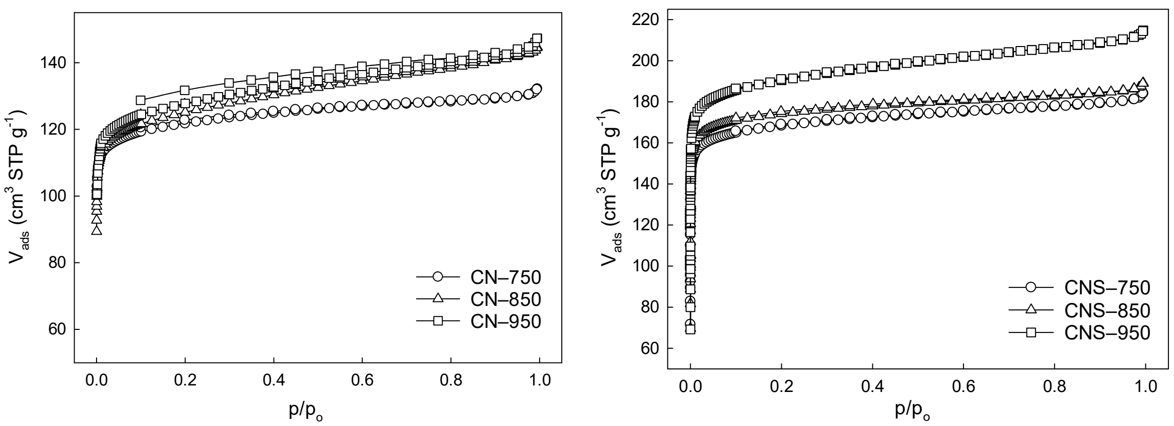

Figure S3. N_2 adsorption-desorption isotherms of the doped carbon gels CN and CNS obtained from a phloroglucinol:aldehyde molar ratio of 1:2 and pyrolyzed at different temperatures.
